# Factors influencing psychotropic prescription by non-psychiatrist physicians in a nursing home for the elderly in Brazil

**DOI:** 10.1590/S1516-31802006000500003

**Published:** 2006-09-07

**Authors:** Florindo Stella, Dorgival Caetano, Jaime Lisandro Pacheco, Elisandra Villela Gasparetto Sé, Acioly Luiz Tavares Lacerda

**Keywords:** Aging, Homes for the aged, Drug prescriptions, Heart diseases, Psychopharmacology, Envelhecimento, Asilos para idosos, Prescrição de medicamentos, Cardiopatias, Psicofarmacologia

## Abstract

**CONTEXT AND OBJECTIVE:**

Although psychotropics are one of the classes of medications most prescribed in nursing homes for the elderly, studies examining prescribing patterns are limited in both number and scope. The present study was undertaken to investigate factors associated with general psychotropic use in a nursing home in Brazil.

**DESIGN AND SETTING:**

Retrospective observational study at the Nursing Home for the Elderly, Institute of Biosciences, Universidade Estadual Paulista.

**METHODS:**

Information on prescriptions was retrieved from the medical records of 108 elderly residents in a nursing home. Sixty-five of these patients, with mean age 74.5 years (± standard deviation 9.4 years), who were taking medications on a regular basis, comprised the sample. The effects of demographic and clinical variables on the psychotropic prescription pattern were examined.

**RESULTS:**

Females were more likely to receive psychotropics (p = 0.038). Individuals on medicines for cardiovascular diseases received psychotropics less frequently (p = 0.001). The number of prescribed psychotropics correlated negatively with both age (p = 0.009) and number of non-psychotropic drugs (p = 0.009).

**CONCLUSIONS:**

Although preliminary, the present results indicated that cardiovascular disease was the clinical variable that most influenced psychotropic prescription. Physicians' overconcern regarding drug interactions might at least partially explain this result. Further investigations involving larger sample sizes from different regions are warranted to confirm these findings.

## RESUMO

**CONTEXTO E OBJETIVO:**

Embora os psicotrópicos sejam uma das classes de medicações mais prescritas em abrigos para idosos, os estudos avaliando o seu padrão de prescrição são limitados em número e escopo. Este estudo visou investigar os fatores associados ao uso de psicofármacos em um abrigo para idosos no Brasil.

**TIPO DE ESTUDO E LOCAL:**

Estudo retrospectivo observacional realizado no Abrigo da Velhice de Rio Claro, Instituto de Biociências, Universidade Estadual Paulista.

**MÉTODOS:**

Dados sobre prescrições foram extraídos dos prontuários médicos dos 108 idosos moradores do abrigo. Sessenta e cinco sujeitos (idade média ± desvio padrão = 74,5 ± 9,4 anos), em uso regular de medicação, constituíram a amostra. Foram examinados os efeitos das variáveis sociodemográficas e clínicas sobre o padrão de prescrição de psicofármacos.

**RESULTADOS:**

As mulheres recebiam mais psicofármacos (p = 0.038); indivíduos em uso de medicações para doenças cardiovasculares recebiam menos psicofármacos (p = 0.001). Houve correlação negativa entre número de psicofármacos prescritos e, ambos, idade (p = 0.009) e número de medicações clínicas (p = 0.009).

**CONCLUSÃO:**

Embora preliminares, os resultados indicam as doenças cardiovasculares como a variável clínica que mais influenciou a prescrição de psicofármacos. Uma excessiva precaução por parte dos clínicos pode explicar parcialmente este resultado. Novas investigações, com amostras maiores e de diferentes regiões são desejáveis para confirmação destes dados.

## INTRODUCTION

As people grow older, disease prevalences rise and medicines for concomitant systemic and central nervous system (CNS) illnesses may be required. The drug-to-drug interaction risk may be further increased by the misuse of psychotropics in nursing homes.^1^ Additionally, age-related biological changes involving both pharmacokinetic and pharmacodynamic parameters are present in the elderly. Decreases in both hepatic metabolism and renal excretion favor the occurrence of drug interaction, especially in the case of substances with prolonged elimination half-life.^2^ In this context, the evaluation of prescribing patterns for psychotropics in nursing homes appears to be of particular interest.

Previous studies have suggested that prescription of psychotropics in nursing homes is importantly influenced by the subject's demographic characteristics, and more so than by diagnostic features.^3,4^ Although psychotropics are one of the classes of medications most prescribed in nursing homes for the elderly, studies examining their prescribing patterns are limited in both number and scope. These studies have reported a wide range of prevalence of psychotropic prescription (10-60%) and have mainly focused on the classes of psychotropics and prevalence of psychiatric morbidity among residents.^5^ Moreover, although the presence of medical illness seems to negatively affect the identification of psychiatric morbidity by general practitioners,^6^ there is to our knowledge no systematic study examining how the prescription of non-psychotropic drugs may influence the non-psychiatrist physician's decision-making process for prescribing psychotropics for the elderly in developing countries.

## OBJECTIVE

The present study was undertaken to investigate factors associated with general psychotropic use in a nursing home in Brazil.

## METHODS

### Subjects

Using a retrospective observational study design, we examined 108 elderly individuals living in a nursing home in the city of Rio Claro, São Paulo, Brazil, that caters for poor elderly individuals living in the region. Of these residents, 65 (60.2%) to whom some kind of drug had been prescribed on a regular basis made up our sample. Thirty-eight residents (58.5%) were on drugs for cardiovascular diseases. To examine the relationship between concomitant use of psychotropics and cardiovascular disease, the subjects were divided into four subgroups: A) 22 with cardiovascular diseases alone; B) 16 with cardiovascular diseases and other clinical diseases; C) 21 with clinical diseases but no cardiovascular problems; D) six with no clinical or cardiovascular disease. The subjects' identities remained undisclosed throughout the stages of the study and all the recommendations of the Helsinki declaration and the local ethical committee were met.

### Data collected

Information on prescriptions, and also clinical and demographic data, was retrieved from the medical records for 2004. With the aim of obtaining a more homogeneous sample, the subjects who were receiving drugs on an "as needed" basis (i.e., without defined posology) were excluded. In accordance with the type of drug prescribed, the subjects were divided into three groups: 1) those taking only psychotropics; 2) those taking only non-psychotropic drugs; 3) those taking psychotropic and nonpsychotropic drugs simultaneously.

### Statistical analysis

Statistical analyses were carried out using SPSS for Windows, version 11 (Chicago, 2002) and a two-tailed statistical significance level was set at 0.05. Analysis of covariance (ANCOVA) was conducted, with the number of non-psychotropic drugs as the dependent measurements, psychotropic use entered as the independent variable, and age as a covariable. The same model was utilized with the chi-squared test to assess the gender effect on psychotropic prescription. Spearman's *rho* correlation coefficients (r) were used to investigate relationships between demographic variables (age) and clinical variables (numbers of psychotropic and non-psychotropic drugs), since their distribution was non-normal (Shapiro-Wilks test).

## RESULTS

The subjects' ages ranged from 58 to 97 years (mean ± standard deviation (SD) = 74.31 ± 9.42). The mean number of drugs taken daily was 3.8.

Although psychotropics were frequently prescribed on a regular basis, neither the diagnoses nor the evaluations by psychiatrists were registered in the medical records. Forty-one subjects (63.1%) were on psychotropics alone, or in combination with non-psychotropic drugs. Eight subjects (12.3%) were on psychotropics exclusively (group 1), with a mean of 2.8 psychotropics taken daily. Among the patients taking psychotropics, 23 subjects were on benzodiazepines, 19 on antipsychotic drugs and 8 on antidepressants, and 16 were taking drugs in other psychotropic classes such as anticonvulsants, anticholinergics and cholinesterase inhibitors. Twenty-four subjects (36.9%) were exclusively on non-psychotropic drugs (mean: 3.7 drugs) (group 2). Thirty-three subjects (50.8%) were concomitantly taking psychotropic and non-psychotropic drugs (mean: 4.2 drugs). Table 1 summarizes the drug prescriptions.

**Table 1:** Daily medications prescribed for 65 residents of a nursing home in Brazil

Groups	n	%	Number of drugs Mean (SD)
**Psychotropics**	8	12.3	2.8 (2.5)
**Non-psychotropics**	24	36.9	3.7 (3.0)
**Psychotropics plus non-psychotropics**	33	50.8	4.2 (4.0)
**Total**	**65**	**100%**	**3.8**

SD = standard deviation.

The subjects on psychotropics (n = 35) received more non-psychotropic drugs than did those not taking psychotropics (3.4 ± 1.54 versus 2.09 ± 1.68; F_1.62_ (F statistic) = 9.98, p = 0.002). Although the subjects not receiving psychotropics were older, this difference did not reach significance (76.43 ± 9.68 versus 72.49 ± 8.94 years, *t* (Student's t test) = 1.52, df (degrees of freedom) = 63, p = 0.093). Female subjects (n = 36) were prescribed psychotropics more often (χ^2^ = 4.298, df = 1, p = 0.038). However, females and males did not differ in terms of quantity of psychotropics (1.0 ± 1.09 versus 1.29 ± 1.15; F_1.62_= 2.12, p = 0.15).

Group A (individuals with cardiovascular diseases alone) took significantly less psychotropics in comparison with the other groups (B = subjects with cardiovascular diseases and other clinical diseases; C = subjects with clinical diseases but no cardiovascular problems): ANCOVA, age as a covariable, F_2.61_ = 7.59, p = 0,001. The subjects in groups A and B did not display a significant difference in relation to the number of psychotropics prescribed (0.73 ± 0.93 versus 0.69 ± 0.79; t = 1.38, df = 36, p = 0.89).

There were negative correlations between both the number of prescribed psychotropics and age (r (Spearman's *rho*) = −0.321, p = 0.009) on the one hand and the number of prescriptions for psychotropic and non-psychotropic drugs (r = −0.58, p < 0.001), on the other. Groups B and C did not differ with regard to age (73.5 ± 9.8 versus 72.22 ± 8.16 years, t = 0.459, df = 41, p = 0.648). However, there was a trend for subjects in group A to be older than those in group C (77.45 ± 10.12 versus 72.22 ± 8.16 years, t = 2.003, df = 47, p = 0.051).

## DISCUSSION

In the present study, nursing home residents with cardiovascular diseases had fewer prescriptions for psychotropics and a greater number of prescriptions for non-psychotropics, as compared with those with other medical diseases. The number of psychotropics prescribed correlated negatively with both age and number of prescriptions for nonpsychotropic drugs. Females were more likely to have psychotropic prescriptions than their male counterparts.

Approximately 38% of the residents were on psychotropics, with a mean of 2.8 drugs taken daily. This is in agreement with studies^3,5^ examining the prevalence of the use of psychotropics among nursing home residents. These studies have reported rates ranging from 27% to 60%, which are higher than the rates of 9.3 to 30.6% found in community studies.^7-9^ Also, other previous studies^10,11^ have reported average numbers of psychotropic drugs prescribed that were similar to what was found in the present study.

Cardiovascular disease is an important risk factor for vascular dementia.^12^ Whether alone or concomitant with other diseases, cardiovascular disease was associated with a smaller number of psychotropics prescribed and seemed to be the clinical condition that most influenced the physician's decision in prescribing psychotropics. This may be partly explained by the fear of pharmacological interactions and lack of familiarity with drugs not usually prescribed by general practitioners. In fact, different psychotropic drugs have been associated with potentially serious cardiovascular side effects, for example QTc interval prolongation on eletrocardiogram.^13^ Alternatively, the issue of underdiagnosis of psychiatric disorders by non-psychiatrists may be more evident among patients with cardiovascular diseases, since these conditions may present neurovegetative symptoms that mimic psychiatric symptomatology (such as loss of energy and appetite, chest pain, increased heart rate, dizziness, paresthesia, memory deficits and insomnia).^14^

Prescribing decisions in nursing homes have raised concerns since evidence supporting them is generally absent.^15^ In the present study, there was a significant negative correlation between the number of prescribed psychotropics and age, which is in line with some previous studies,^5^ but not all.^16,17^ This finding appears to be counterintuitive, since some authors have reported a higher prevalence of mental disorders in the elderly.^5^ This might be explained by the fact that mental disorders are often underdiagnosed and, consequently, undertreated among older people.^18^ Alternatively, overconcern regarding the use of psychotropics among older residents and possible interactions involving psychotropics could at least in part account for this finding. The negative correlation between the numbers of psychotropic and non-psychotropic drugs observed in the present study supports the latter hypothesis.

More women were prescribed psychotropics than men, which is in agreement with some previous studies.^19,20^ but not all.^21^ This may be due to a variety of reasons. Firstly, except for some specific diagnoses, females are more likely to have psychotropics prescribed.^19,22^ Secondly, caregivers' attitudes towards behavioral disorders and the physician's ability to diagnose and treat psychiatric disorders may also be biased according to gender.^23^ Finally, gender differences in coping with and expressing psychological suffering might at least partially account for this finding.^24,25^

The present findings should be interpreted with caution since several limitations were present. Firstly, it was an uncontrolled retrospective study conducted in one institution in a city in the interior of Brazil, where access to continuing medical education is limited. Secondly, since only a few individuals had diseases other than cardiovascular disease (such as cerebrovascular diseases, Parkinson's disease, dementia, lung diseases or bone-joint diseases), these conditions were classified together as "other diseases", which thus produced a very heterogeneous group. Finally, the sample size was limited. This latter observation is especially valid with regard to the analyses involving subgroups.

## CONCLUSIONS

To our knowledge, this is the first study evaluating the prescription of psychotropics by non-psychiatrist physicians in a nursing home for the elderly in Brazil. Although preliminary, the present results indicate that females were more likely to have psychotropics prescribed, and that age was negatively associated with the number of psychotropics prescribed. Moreover, the presence of cardiovascular disease negatively influenced the prescription of psychotropic medications. General practitioners' overconcern regarding drug interactions when prescribing psychotropics might at least in part explain this finding. Further investigations involving nursing homes from different regions of Brazil are warranted, to explore the reasons for these associations and to test whether the present results can be generalized.
